# Impacts of patient characteristics on hospital care experience in 34,000 Swedish patients

**DOI:** 10.1186/1472-6955-11-8

**Published:** 2012-06-14

**Authors:** Axel Wolf, Lars-Eric Olsson, Charles Taft, Karl Swedberg, Inger Ekman

**Affiliations:** 1 Institute of Health and Care Sciences, Sahlgrenska Academy, University of Gothenburg, Gothenburg, Sweden; 2 Department of Molecular and Clinical Medicine, Sahlgrenska Academy, University of Gothenburg, Gothenburg, Sweden; 3 Centre for Person-Centred Care (GPCC), University of Gothenburg, Gothenburg, Sweden

**Keywords:** Patient-reported outcome, Self-rated health, Functional status, Frail, Care experience, Care disparity, Patient-centred care

## Abstract

**Background:**

Standardized patient surveys are widely used for assessing quality of healthcare from the patient perspective. An important purpose of such surveys is to identify disparities in care among different patient groups. The purpose of this study was to 1.) evaluate aspects of the validity of the adapted Swedish version of the Picker Patient Care Experience -15 (PPE-15) survey and 2.) examine the explanatory value of various socio-demographic and health characteristics in predicting patients’ care experiences.

**Methods:**

A retrospective cross-sectional study design was used. Patients discharged from internal medicine wards at regional and university hospitals in different parts of Sweden during 2010 were invited to participate in the regularly administered national care-experience survey for hospital care. The internal validity of the PPE-15 was assessed with Cronbach’s alpha and item-scale correlations. Pearson product–moment correlation coefficients were used to compare PPE-15 total scores with overall care satisfaction ratings and Spearman correlation coefficients were used to compare PPE-15 total scores with various patient characteristics. Multiple linear regression analysis was performed to examine the influence of various patient characteristics on PPE-15 scores.

**Results:**

The response rate was 66% (n = 34 603). Cronbach’s alpha was 0.87. The correlation between the PPE-15 total score and overall care satisfaction was high (0.62, p < 0.0001). Good self-rated health (SRH) and having Swedish as native language were associated with better care experiences and poorer experiences with greater healthcare utilization, higher age, functional impairment and being female. All examined characteristics, except language, were significant predictors in the regression model and SRH was the strongest predictor; however, the model explained only 7% of the total variance. Vulnerable patients (i.e. poor SRH and functional impairment) reported significantly less positive care experiences than did non-vulnerable patients (mean PPE-15 score 75 vs 85; p < 0.0001).

**Conclusions:**

Our results supported the internal validity of the Swedish adapted version of the PPE-15. The explanatory value of the examined patient socio-demographic and health characteristics was low, suggesting the need for exploring other patient-related determinants of care experiences. Our findings also suggest a care paradox: patients in greatest need of hospital care are least satisfied with the quality of the care they receive.

## Background

The importance of incorporating the patient perspective in evaluations of the quality of health care has long been emphasized by international organisations such as the Organization for Economic Cooperation and Development (OECD) and World Health Organization (WHO) [1,2]. To this end, a number of standardized patient surveys have been developed and are widely used today for eliciting patients’ assessments of health care quality [3].

Results from patient surveys may serve to stimulate and guide quality improvement initiatives by indicating specific areas where efforts and resources should be targeted. However, interpretation and application of patient survey results are not straightforward. Although patient ratings may reflect directly on factors amenable to control and change by health care services, such as care structures and processes, they may also be determined by personal characteristics of respondents [4]. In fact, a large body of research has identified relationships between patient characteristics such as age [5], gender [4], socioeconomic status [4], health status [6], etc. and experiences of or satisfaction with health care. Knowledge gained from this research has implications not only for quality improvement aims of providing equitable health care [7], but also for interpretation of survey results by disentangling the effects of immutable patient characteristics from more mutable health care delivery factors.

One of the most widely used patient surveys during the last 20 years is the Picker Institute’s hospital survey. It has proven useful internationally for measuring patients' hospital experiences for the purpose of benchmarking care quality [8]. In Sweden, an adapted version of the Picker survey is currently used to assess patient experiences on a national level; however, to date the Swedish version has not been psychometrically evaluated.

The objective of this study was two-fold: 1.) to evaluate aspects of the internal validity of an adapted Swedish version of the Picker PPE-15 survey and 2.) to explore the explanatory value of various socio-demographic and health characteristics in predicting patients’ care experiences (PPE-15 total score).

## Method

### Design, patients and setting

This retrospective cross-sectional study analyses parts of a national patient care experience survey organised by the Swedish Association of Local Authorities and Regions during 2010. The survey population included 56000 adults (16 years or older) who had been admitted overnight to Swedish internal medicine wards at both small county and large university hospitals.

The survey was administered by post following an adapted version of Dillman's Total Design Method [9-11], used in previous Picker surveys [8] Briefly, the survey was mailed to patients one month after their discharge from hospital, together with an informed consent cover letter explaining the purpose of the survey and a self-addressed stamped return envelope. A reminder with a new survey was sent out after three weeks and, if necessary, again after seven weeks.

The study was approved by the Regional Ethical Review Board of the University of Gothenburg and the study conforms to the principles outlined in the Declaration of Helsinki.

### The survey instrument

The original Picker institute “in-patient” survey consists of 40 items covering eight dimensions: information and education, coordination of care, emotional support, respect for patient preference, physical comfort, involvement of family & friends, and continuity & transition and overall impression [12]. Rather than asking the patient questions about general care satisfaction, the items ask how the patient perceives specific areas of his/her care (e.g., Did you want to be more involved in decisions made about your care and treatment?).

In 2002, the Picker Institute developed and validated a 15-item version (PPE-15) of the original “in-patient” survey [12]. The PPE-15 is considered to represent a universal set of items that constitute a core set of questions that are applicable for the majority of patients. Items are rated on three- or five-point Likert-type scales. In the original PPE-15, ratings are dichotomized (presence or absence of a problem) and summed and transformed to produce a “problem score”, ranging from 0 (no problems) to 100 (rating all items as problems).

A Swedish adapted version of the original Picker “In-patient survey” has been used previously [8,13]. Since then, the Swedish version has been further developed during the last 10 years and currently contains 63 items. For purposes of this study the 15 items that comprise the PPE-15 were extracted in order to compute the PPE-15 total score (table 1).

**Table 1:** The PPE-15 items divided into the seven Picker domains

**Domain and item (n)**	**PPE-15 r (p-value)**
**Information and education** (n = 29248)	0.73 (<0.0001)
***Item 1****(n = 26760): When you had important questions to ask a doctor, did you get answers that you could understand?*	0.65 (<0.0001*)*
***Item 2****(n = 27903): When you had important questions to ask a nurse, did you get answers that you could understand?*	0.60 (<0.0001)
**Coordination of care** (n = 29424)	0.47 (<0.0001)
***Item 3****(n = 29424): Sometimes in a hospital, one doctor or nurse will say one thing and another will say something quite different. Did this happen to you?*	0.47 (<0.0001)
**Emotional comfort** (n = 22863)	0.82 (<0.0001)
***Item 4****(n = 19349): If you had any anxieties or fears about your condition or treatment, did a doctor discuss them with you*	0.71 (<0.0001)
***Item 8****(n = 21090): If you had any anxieties or fears about your condition or treatment, did a nurse discuss them with you?*	0.68 (<0.0001)
***Item 9****(n = 27959): Did you find a doctor/nurse to talk to about your concerns?*	0.71 (<0.0001)
**Respect patient preference** (n = 29555)	0.79 (<0.0001)
***Item 5****(n = 29459): Did the staff talk in front of you as if you weren’t there?*	0.34 (<0.0001)
***Item 6****(n = 29131): Did you want to be more involved in decisions made about your care and treatment?*	0.70 (<0.0001)
***Item 7 (****n = 29465): Overall, did you feel you were treated with respect and dignity while you were in hospital?*	0.64 (<0.0001)
**Physical comfort** (n = 17410)	0.57 (<0.0001)
***Item 10****(n = 17410): Were you ever in pain, if yes; Do you think the hospital staff did everything they could to help control your pain?*	0.57 (<0.0001)
**Involvement of family and friends** (n = 23188)	0.57 (<0.0001)
***Item 11****(n = 17725): If your family or someone else close to you wanted to talk to a doctor, did they have enough opportunity to do so?*	0.55 (<0.0001)
***Item 12****(n = 16474): Did the doctors or nurses give your family or someone close to you all the information they needed to help you recover?*	0.52 (<0.0001)
**Continuity and transition** (n = 21306)	0.77 (<0.0001)
***Item 13****(n = 21867): Did a doctor explain the purpose of the medicines you were to take at home in a way you could understand?*	0.63 (<0.0001)
***Item 14****(n = 19045): Did a doctor tell you about medication side effects to watch for when you went home?*	0.57 (<0.0001)
***Item 15****(n = 24338): Did someone tell you about danger signals regarding your illness or treatment to watch for after you went home?*	0.62 (<0.0001)

Pearson product-moment correlations between the PPE-15 score and Picker domains/items without correction for overlap are presented.

The Swedish version differs slightly from the original PPE-15 in relation to two items: item 5 “Did the *doctors* talk in front of you as if you weren’t there?” and item 9 “Did you find *someone* on the hospital staff to talk to about your concerns?” In the Swedish version “doctors” (item 5) were replaced by “the staff” and “someone”(item 9) replaced by a “nurse/doctor”. The Swedish version also uses a more refined scoring procedure than the original PPE-15. Briefly, response alternatives are coded such that 0 = problem, 0.5 slight problem and 1 = no problem and summarized into a total and dimension scores ranging from zero (scoring all items as problems) to 100 (no problems).

### Study variables

PPE-15 total scores were used in the analyses. Other study variables comprised all patient characteristics included in the Swedish national survey, namely gender, age groups (<= 44 years, 45-64 years, 65-74 years, > = 75 years), education level (Elementary/High school/University), native language (Swedish/other), healthcare utilisation within the previous six month (Never, once, 2-3 times, > = 4 times), self-rated health (SRH; excellent/very good/good/fair/bad), functional impairment (need for assistance to and from the bathroom/or bedpan) and overall satisfaction with care or treatment (“Overall, how satisfied are you with the care/treatment you received”; 5-point scale from excellent/very good/good/fair/bad). In the sub-group analysis, an indicator variable was created to indicate patients who were vulnerable versus non-vulnerable based on combined SRH and functional impairment ratings [14-16]. Specifically, patients who rated their health status as excellent, very good or good and reported no functional impairment were coded as non-vulnerable, whereas vulnerable patients were those with SRH ratings fair or bad and reported functional impairment.

### Statistical analyses

The internal validity of the Swedish PPE-15 was evaluated with Cronbach´s alpha and by estimating item-to-scale correlations using Pearson product–moment correlations, correcting for overlap. Bivariate analyses of associations between patient characteristics and PPE-15 total scores were examined with the Mann–Whitney *U*-test for dichotomised variables and the Spearman correlation coefficient for ordinal variables.. Multiple linear regression analyses were performed to determine the influence of patient characteristics (dependent variables) on PPE-15 scores. To estimate the effect size of the differences in the PPE-15 total score between groups (vulnerable vs. non-vulnerable), Cohen’s *d* was calculated (M_2_-M_1_/pooled SD) [17]. The magnitude of the effect sizes may be judged against the criteria suggested by Cohen: trivial (0 to <0.2), small (≥0.2 to <0.5), moderate (≥0.5 to <0.8) and large (≥0.8). The half-scale method was applied to impute missing item values in all analyses of associations between patient characteristics and the PPE-15 total score. All statistical tests were two-sided with a significant level of P ≤ 0.01. The data were analysed using SAS version v9.2 (SAS Institute Inc., Cary, NC, USA).

## Results

A total of 34 603 patients returned evaluable surveys (response rate 66%). The majority of respondents were 65 years old or above (59%) and native speakers (90%), 44% of the respondents were males and 50% of the respondents had attended higher education (High school (31%) or University (19%). Overall, 62% of the respondents had utilised some healthcare services within 6 month of the present hospitalisation and half of the respondents (54%) reported SHR as excellent, very good or good. Reasons for non-response could be established in 5126 cases: refusal (n = 941), severe illness (n = 1281), death (n = 1951), language problems (n = 25), wrong postal address (n = 590) and screening failure (n = 338).

The mean PPE-15 score was 81.1 (SD = 17.6, mdn 85.0, IQR = 71.4- 95.4). In total, 19% scored at the ceiling (PPE-15 = 100) and 0% scored at the floor.

### Reliability and validity

The internal consistency reliability was satisfactory (Cronbach’s alpha = 0.87). Item-to-total PPP-15 score correlations, corrected for overlap, ranged from r = 0.26 (item 5) to r = 0.69 (item 9). Domain to total score and item to total score without correction for overlap are presented in table 1.

The correlation between PPE-15 total scores and overall satisfaction with care/treatment was high (0.62, p < 0.0001).

The adapted Swedish PPE-15 scoring method had a high correlation with the original PPE-15 scoring method (r = -0.94, p < 0.0001).

### Care experience and patient characteristics

Of the 32 517 patients included in the analysis, 46% of the responding patients were males and 61% of the patients were older than 65 years (Table 2). PPE-15 total scores correlated significantly with SRH (r = 0.24, p < 0.0001), age (r = -0.03, p < 0.0001), healthcare utilisation (r = -0.06, p < 0.0001). Educational level (r = -0.005, p = 0.38) had no significant correlation with the PPE-15 score. Significant differences in PPE-15 scores and the dichotomous variables were found between functionally impaired patients and non-impaired patients (M = 79.7 vs. 82.5, p < 0.0001), male vs. female patients (M = 82.1 vs. 80.2, p < 0.0001) and patients with Swedish as their native language vs. non-native speakers (M = 81.2 vs. 80.0, p = 0.004).

**Table 2:** Patient characteristics divided into total population and vulnerable vs. non-vulnerable patients

**Variable**	**Total population**	**Vulnerable**	**Non-vulnerable**	**p-value**
	**n (%)**	**n (%)**	**n (%)**	
Gender				
Female	16738 (54)	4086 (59)	4339 (47)	<0.0001
Age class				
<= 44 years	4162 (13)	387 (5)	1748 (18)	
45-64 years	8376 (26)	1438 (20)	2811 (30)	
65-74 years	8150 (26)	1637 (23)	2630 (28)	
> = 75 years	11086 (35)	3557 (51)	2275 (24)	<0.0001
Healthcare utilization within last 6 month				
Never	6362 (20)	634 (9)	2763 (29)	
Once	5042 (16)	779 (11)	1848 (20)	
2-3 times	9672 (31)	2277 (33)	2707 (29)	
> = 4 times	10493 (33)	3282 (47)	2111 (22)	<0.0001
Native language				
Yes	29348 (92)	6426 (91)	8880 (94)	<0.0001
Education				
Elementary school	15169 (48)	4156 (60)	3763 (40)	
High school	10075 (32)	1839 (26)	3338 (36)	
University	6111 (19)	948 (14)	2201 (24)	<0.0001
Self-rated health				
Excellent	3243 (10)	0 (0)	1505 (16)	
Very good	10873 (34)	0 (0)	3102 (32)	
Good	9096 (29)	0 (0)	4944 (52)	
Fairly good	5787 (18)	5271 (74)	0 (0)	
Poor	2747 (9)	1831 (26)	0 (0)	<0.0001
Functional impairment				
Dependence	15290 (48)	7102 (100)	0 (0)	
Independence	16594 (52)	0 (0)	9551 (100)	<0.0001

For categorical variables n (%) is presented. For comparison between groups Fisher´s Exact test was used for dichotomous variables and the Mantel-Haenszel Chi Square test was used for ordered categorical variables.

All patient variables were entered stepwise into the multiple linear regression model. All variables except native language were significant predictors of PPE-15 scores. SRH and functional impairment were the strongest predictors in the model. The model explained 7% of the variance in PPE-15 scores (R^2^ = 0.07, p < 0.0001) (table 3).

**Table 3:** Independent predictors of PPE-15 total score

	**Parameter Estimate**	**SE**	**Adjusted p value**
Subjective health (bad health-good health)	4.37	0.10	<0.0001
Functional impairment (dependence, independence)	2.24	0.21	<0.0001
Gender (male, female)	-1.31	0.21	<0.0001
Age	0.96	0.11	<0.0001
Highest education level	-1.01	0.14	<0.0001
Healthcare utilization last six month	0.47	0.10	<0.0001

Multiple linear regression analysis including all patient characteristics. R^2^ =0.07.

### Care experience in vulnerable patients

**Figure 1:**
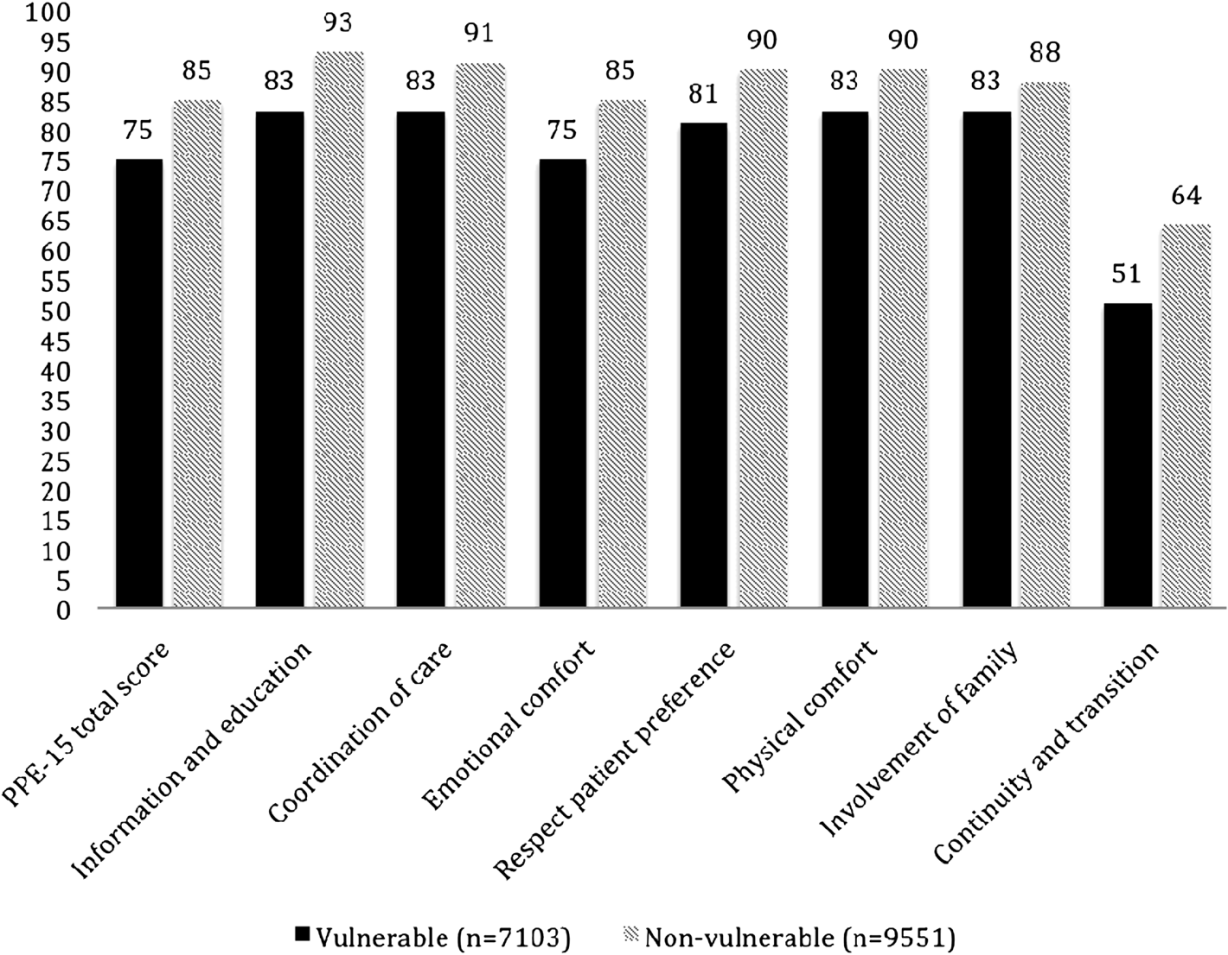
**Care experience illustrating vulnerable vs. none-vulnerable patients.** PPE-15 total score and domain score between vulnerable and non-vulnerable patients (ps < 0.0001).

Vulnerable patients (n = 7103) had significantly poorer PPE-15 total scores (M 75, SD 19.8) than non-vulnerable patients (n = 9551) (M 85, SD 15.0, p < 0.0001) (Figure 1). The effect size of this difference was moderate (Cohen's *d* = 0.58). Significant differences were found between the groups in all seven dimensions (ps < 0.0001) (Figure 1), and effect sizes ranged from small (Cohen's *d* = 0.22; Involvement of family and friends) to moderate (Cohen's *d* = 0.52; Respect patient preference).

There were significant differences in patient characteristics between the groups (table 2). For example, the vulnerable patients were older and had utilised more healthcare resources prior to the present hospitalisation than the non-vulnerable patients (p < 0.0001) (table 2).

## Discussion

Our results generally support the internal validity of the Swedish adapted version of the PPE-15. Construct validity was supported by a high correlation between general care satisfaction ratings and PPE-15 scores. Patient characteristics such as self-rated health and functional impairment had the highest relationship with the PPE-15 total score. The explanatory value of the examined patient socio-demographic and health characteristics was low, suggesting the need for exploring other patient-related determinants of care experiences such as previous hospital experiences/expectations of care and self-efficacy. Vulnerable patients, defined as having poor SRH and some degree of functional impairment, reported their care experience significantly less positive when compared with healthier and less functionally impaired patients.

Our results for the Swedish adapted version were similar to those reported for the original Picker PPE-15 [12] regarding internal consistency reliability (Cronbach alpha =0.87 vs 0.80). Interestingly, in the original PPE-15 the item-total score correlation of item 5 (staff talking as if I wasn´t there) was below generally accepted limits (r < 0.30), and in the Swedish version it also had the lowest correlation. The item remained in the original PPE-15 survey because the removal of the item did not appreciably improve alpha [12], yet it could be questioned if the item should be retained in the PPE-15.

Benchmarking care experiences is increasingly prioritized by governmental agencies, and may in the future become an important evaluation tool for patients when comparing treatment and care options [18]. National care experience surveys are important tools for assessing and monitoring healthcare quality and the widespread international usage of the Picker in-patient survey also enables countries to compare themselves on an international level as well.

The present study demonstrated an association between care experience and patient characteristics such as SRH and functional impairment. Studies have previously shown an association of SRH and physical function with mortality and morbidity [19,20]. Particular emphasis has been placed on investigating relationships between general health and clinical outcomes [21-23]. Consequently, greater focus has been put on patient-reported outcomes as recognised by the European Medicines Agency (EMA) [24] and The Patient-Centered Outcomes Research Institute in the USA [18]. Our findings showed that SRH had the highest impact on the patients’ care experience. In this study demographic characteristics such as age had a weak association with care experience. While previous studies have suggested an association between increasing age and higher care satisfaction [4], it has also been suggested that this association declines in patients above 80 years [6]. Overall, we found that various socio-demographic and health characteristics could explain merely seven percent of the variance in the PPE-15 scores. This shows the complexity of measuring care experience, and suggests that external factors may have a greater impact on care experiences than socio-demographic and health characteristics. For the patient, hospitalisation involves a constant interaction and adjustment to the structures of the care environment, including the attitudes and belief systems of the nurses, physicians and other staff that work there. It has been suggested that health care professionals adapt their communication style and care strategies to the social, emotional and physiological characteristics of their patients [25,26]. An important finding was that vulnerable and non-vulnerable patients differ substantially in their experiences of the quality of their care. The mean 10-point difference between the two groups may also be relevant in term of the effect size (PPE-15 total score Cohen's *d* = 0.58). Healthcare professionals may view vulnerable patients as having more complex and demanding support needs and hence may give priority to meeting such needs at the expense of other aspects of quality care, such as involving patients in care decision making. Previous research has shown that patients who demonstrate more rapid adaptation to the present care structures, that are perceived as more satisfied and more adherent receive more information, empathy and are more involved in care decisions [27]. A cause of particular concern in the present study is that vulnerable patients, who were older and utilized more healthcare resources, reported worse care experiences than the non-vulnerable patient. Therefore, care experience may be seen as a continuum of events, hence factors such as the patient’s previous hospital experiences/expectations of care, self-efficacy level and notion of shared decision making may constitute important aspects in evaluating care experience that should be further explored. This is particularly relevant in chronic conditions where care efforts often focus primarily on providing support in self-management. Although these factors are not directly healthcare quality indicators, indirectly they reflect the effectiveness of the support programs offered the patient and should therefore be regularly monitored.

Effective care is widely considered a collaborative process between all concerned stakeholders, and in particular the patient [28]. Care communication and patient participation are part of a complex system which requires co-operation and respect between the patients and caregivers [29]. As such this collaborative process should be built upon increased patient participation and dialog, in order to find common ground and understanding about the direction and goals of the care. One example of the difficulty of health care providers to communicate with patients is the marked discrepancy between the perceptions of patients and the physicians regarding the content of treatment information [30]. Although the patients understood the treatment information, they still needed guidance and advice on how to follow the treatment plan. The physicians, on the other hand, assumed that the patients’ non-adherence to the treatment plan was due to the difficult nature of the information. In the present study, the lowest care experience scores for both vulnerable and non-vulnerable patients were seen in the same domain, namely continuity and transition (Figure 1). Overall. it seems that quality hospital care experience from the patients’ view, regardless of the patient’s health or functional status, is one that supports the patient throughout the entire care continuum, both at the hospital and in the transition back to the patient’s home.

### Study limitations

Our study has several limitations. Our classification of functional impairment was based on patients’ reports of need for assistance when toileting. The present study was not designed to determine the actual cause of the patients’ need for assistance when toileting (e.g., mobile surveillance, urine catheters or impaired functional status) and space and logistic restraints in the survey prohibited more comprehensive assessments of physical impairment. Nevertheless, being dependent on someone for help with toileting should reflect physical impairment and may be an easy and practical way of assessing functional impairment.

The associations between various socio-demographic and health characteristics were significant yet low and given the cross-sectional nature of this study, no conclusions can be drawn regarding causality. Furthermore, owing to the large size of the survey sample, small correlations were still statistically significant; hence, the clinical significance of the reported correlations is unclear.

## Conclusions

The Swedish adapted version of the PPE-15 can be used as a valid benchmarking tool for care experience. Patient characteristics such as self-rated health and functional impairment had the highest associations with patient’s care experience. The explanatory value of the examined patient socio-demographic and health characteristics was, however, low. Our findings indicate a care paradox: hospital care, as delivered by care professionals in Sweden, provides care experiences which to a higher degree acknowledge the needs and resources of independent patients in good health, but does not provide equally satisfactory care for vulnerable patients.

## Competing interests

The authors declare that they have no competing interests.

## Authors’ contributions

AW: Study design, data collection, data analysis, preparing the manuscript. L-EO: Study design, data analysis, preparing the manuscript. CHT: Preparing the final manuscript. KS: Study design, preparing the final manuscript. IE: Study design, preparing the final manuscript. All authors read and approved the final manuscript.

## Pre-publication history

The pre-publication history for this paper can be accessed here:

http://www.biomedcentral.com/1472-6955/11/8/prepub
